# Response: A commentary on “*Eucalyptus obliqua* seedling growth in organic vs. mineral soil horizons”

**DOI:** 10.3389/fpls.2016.00052

**Published:** 2016-02-04

**Authors:** Karen M. Barry, David P. Janos, David M. J. S. Bowman

**Affiliations:** ^1^Tasmanian Institute of Agriculture and School of Land and Food, University of TasmaniaHobart, TAS, Australia; ^2^Department of Biology, University of MiamiCoral Gables, FL, USA; ^3^School of Biological Sciences, University of TasmaniaHobart, TAS, Australia

**Keywords:** *Eucalyptus obliqua*, fire, regeneration, forest management, nutrition

Our recent paper (Barry et al., 2015) sought to understand factors limiting the growth of *Eucalyptus obliqua* seedlings in temperate native forest regeneration. A commentary from Neyland and Grove (2015) followed, that stated that while the main focus of our paper was not contentious, they disagreed with our concluding “Forest Management Implications” section. This provides an opportunity to expand and clarify some points in our original article. In disagreeing, Neyland and Grove (2015) cited several empirical studies that have shown “…  the fundamental importance of burnt soil as part of the regeneration cycle … ”. We do not dispute those studies. Instead, the intent of our work was to move toward a mechanistic understanding of biological processes. That is, why do seedlings fail to establish on unburnt litter? We aimed to discern factors—particularly mineral nutrition and soil–fungi interactions—related to eucalypt establishment that might help to explain which components of fire regeneration are critical and which are not. We used a pot-based study to attempt to examine these factors in isolation. Using pot-trial results as a basis to extrapolate to forest ecological processes is well-established in a large volume of scientific literature.

Neyland and Grove (2015) state that we confounded the term *stocking* with *seedling density* when interpreting the results of Neyland et al. (2009). The section of Neyland et al. (2009) on which we based our comments stated “Of the coupes burnt at lower intensities, only WR1B, which had a very poor burn but a very high rate of natural seedfall, achieved a commercially acceptable seedling density.” While the density was lower than those coupes with high intensity burns, the seedling density did reach the “desired commercial minimum” stated as 2500 stems ha^−1^, by year 3 (Neyland et al., 2009). We interpreted this result as showing that in spite of its poor burn, under some suite of conditions which prevailed in coupe WR1B, seedling density could reach the “levels considered necessary for future development of a productive regrowth eucalypt forest,” not that it always would do so. We agree that the results of this one coupe alone are not sufficient to suggest that non-burn alternatives will be commercially viable, however it does provide a biological basis for further study.

Neyland and Grove (2015) expressed aversion to our suggestion of removal of woody debris in order to control competing vegetation, which is likely inhibitory to regenerating eucalypts. They used the acronym “CWD” which refers to coarse woody debris. We did not suggest removal of CWD or all woody debris. Our mention of “repeated re-clearing” meant clearing (cutting) of living material to reduce competition, in the same way that “clearfell” is the process of harvesting, which is followed by debris management. We submit that mechanical methods to remove debris could be trialed and designed for retention of the same piece size-distribution that occurs with best-practice burning for habitat conservation and also to ensure that the full suite of understory plant species return. Whether, re-clearing to minimize competition could be managed practically and economically is a separate issue from whether it is a biologically feasible alternative to burning that might achieve similar regeneration outcomes. Investigating alternatives to burning (even those likely to be unprofitable) in field trials would help expand understanding of the biological limits to eucalypt seedling growth and might reveal alternatives that are both environmentally and economically sustainable.

Alternatives to burning are being trialed for fuel reduction management, and a recent announcement by the Australian federal government of a funded program to explore new forest fire fuel reduction methods has been supported by the (Australian Forest Productions Association (AFPA), 2015). This trial, which will examine bushfire prevention through mechanical fuel removal across Victoria, is based on studies in California that have shown “mechanical methods are not causing ecological harm… they're actually doing some real ecological work and sometimes doing things economically” (Grindley, 2015). It will demonstrate to what degree the Californian experience is applicable to southern Australia and the trials will have relevance to native forest harvesting.

There is significant public interest in ensuring best practice forest management is undertaken in Tasmania, such that the Regional Forest Agreement (Commonwealth of Australia State of Tasmania, 1997) led to programs seeking alternatives to clearfell, burn and sow (CBS). CBS silviculture involves extreme mechanical disturbance, and is predicted to lead to species losses of forest flora and fauna (Baker and Read, 2011). While variable retention was investigated as an alternative to clearfell (Forestry Tasmania, 2009), alternatives to burning have not been investigated, despite emerging understanding of potential public health risks associated with smoke exposure (Henderson and Johnston, 2012; Johnston et al., 2012). Reduction of post-logging burning would reduce greenhouse gas emissions and enable some woody residues to be used for energy production (Bradshaw et al., 2013). Past plans in Tasmania to produce electricity from wood waste were prevented because residues were not included in Australia's Renewable Energy Target (RET). However, residues from native forests harvested for high-value solid timber are now accepted as biomass fuels under the revised RET (Brown and Coote, 2015). A pertinent model exists in Sweden, where about one-quarter of domestic energy production is from forest-based bioenergy and the ash thereby produced is returned to harvested sites to help restore the mineral nutrient balance (Levin and Eriksson, 2010). The potential for use of renewable forest biomass for energy in Tasmania was favorably assessed by Rothe (2013).

While we acknowledge the research on eucalypt silviculture conducted by the forest industry to improve biodiversity conservation and sustainable outcomes, we hope to stimulate further investigations of eucalypt regeneration biology so that alternative silvicultural practices can be explored in field trials in the near future. We contend that the biological limitations of eucalypt regeneration should be understood independently of economic viability of alternatives, even though the latter will determine forestry practice.

## Author contributions

KB wrote the commentary. DB supplied several references and points of interest to include. DJ edited the commentary.

### Conflict of interest statement

The authors declare that the research was conducted in the absence of any commercial or financial relationships that could be construed as a potential conflict of interest. The reviewer Mark Andrew Hunt declares that, despite being affiliated with the same institute as the authors Karen M. Barry and David M. J. S. Bowman, the review process was handled objectively.
